# Bridging: Accelerating Regulatory Acceptance of Reduced-Risk Tobacco and Nicotine Products

**DOI:** 10.1093/ntr/ntac041

**Published:** 2022-02-16

**Authors:** Marianna Gaca, Justine Williamson, Helena Digard, Louise Adams, Lauren Hawkridge, Christopher Proctor

**Affiliations:** British American Tobacco (Holdings) Ltd, Globe House, 4 Temple Place, London WC2R 2PG, UK; British American Tobacco (Holdings) Ltd, Globe House, 4 Temple Place, London WC2R 2PG, UK; Research and Development, British American Tobacco (Investments) Ltd, Regent’s Park Road, Southampton SO15 8TL, UK; Research and Development, British American Tobacco (Investments) Ltd, Regent’s Park Road, Southampton SO15 8TL, UK; Research and Development, British American Tobacco (Investments) Ltd, Regent’s Park Road, Southampton SO15 8TL, UK; DoctorProctorScience Ltd, 157 Cavendish Meads, Ascot, Berkshire SL5 9TG, UK

## Abstract

**Introduction:**

The number and variety of alternative tobacco and nicotine products that can potentially provide reduced-risk choices for cigarette smokers who switch completely to such products instead of continued smoking have grown substantially in the past decade. Innovation and choice are likely to improve the prospects of smokers making the switch, but this provides challenges to regulators and manufacturers to ensure that changes to regulations and products promote and do not hinder contributions to tobacco harm reduction.

**Aims and Methods:**

This paper looks at where bridging data sets for tobacco heating products, closed system vaping products, and oral nicotine products might enable innovation while protecting the interests of consumers.

**Results:**

We review product data from chemical studies and a toxicological study showing how bridging can be applied and consider what product development changes might allow bridging from existing datasets or trigger the need for new ones.

**Conclusions:**

Bridging across specific product ranges can increase the speed of innovation, foster competition, and limit the burden of assessment for regulators while maintaining product safety and quality.

**Implications:**

Bridging partial data sets is an established practice within other industries, that aims to improve efficiency with regulatory approvals, accepts natural product variation, and supports product innovation. We review product data from chemical studies and a toxicological study showing how bridging can be applied and consider what product development changes might allow bridging from existing datasets or trigger the need for new ones. This in turn can increase the speed of innovation, foster competition, and limit the burden of assessment for regulators while maintaining product safety and quality.

## Introduction

Alternative tobacco and nicotine products offer an opportunity to substantially change the projected public health impact of cigarette smoking, but only if the products are demonstrated to reduce risk substantially as compared to continued smoking and are good enough to provide smokers a satisfactory complete alternative to smoking conventional cigarettes.^1,2^ Changes in the population dynamics of tobacco and nicotine product use have been seen in some countries since the introduction of electronic nicotine delivery systems (ENDS), particularly vaping products,^3^ as was seen in Sweden with increased use of snus decades ago.^4^ This change has perhaps been most notable in the United Kingdom, where a considerable number of smokers have switched entirely to vaping,^3^ this is likely to be in part due to strong public health support in this country for tobacco harm reduction approaches. Unlike snus, early versions of vaping devices were relatively complicated and posed a variety of barriers to adoption by smokers. A study by McKegany and Dickson looking at why more smokers had not switched to e-cigarettes found that many smokers disliked the technology, the chemical nature of the e-liquids, and the complexity of the devices.^5^ However, innovation in formats, devices, and flavors, from small and medium-sized enterprises, as well as multinational companies, have improved the products, and the rate of innovation continues to increase as more companies enter into the field of alternative tobacco and nicotine products.^6,7^

The concept of tobacco heating products (THPs) (known originally as heat-not-burn tobacco products) as a way to reduce toxicant generation was introduced in the 1980s^8^ but was unsuccessful with smokers.^9,10^ Technological advances in micro-electronics and batteries have made THPs more recently potential alternatives to cigarettes, and this new generation of products are found in a wide range of product designs and temperatures applied to the tobacco, but with a common design feature of not having self-sustained exothermic combustion of the tobacco.^11^

The very recent introduction of oral nicotine products (NPs) as an alternative to cigarette smoking has seen a rapid adoption particularly in some countries with a tradition of oral tobacco use being seen as “less harmful” than tobacco products.^12^

Regulators are rightly cautious about the introduction of new tobacco and nicotine products because there are no long-term epidemiology data indicating what risks they may carry and whether they may serve as initiators of cigarette smoking.^13^ Moreover, nicotine is addictive and any product that serves as an alternative for smokers will likely deliver similar amounts of nicotine as cigarettes.^14,15^ Some regulators do not accept the need for alternative products, believing that traditional tobacco control methods are working sufficiently to reduce smoking rates, while others believe a harm reduction approach including introduction of alternative consumer products would accelerate goals of reducing projected tobacco-related mortality and morbidity. This has led to wide-ranging regulatory environments, from prohibition to product restrictions (eg, nicotine content and ingredients) to the introduction of product standards that define categories.^3^ As the eighth report of the WHO Study Group on Tobacco Product Regulation noted, regulatory approaches to the alternatives are very diverse between countries.^16^

The challenge for any regulator that wishes to encourage smokers to switch from smoking to alternative products, and particularly with ENDS, is the very large set of diverse products that have been placed on the market. The US FDA’s Center for Tobacco Products has had to evaluate millions of individual products following regulations that deemed ENDS to be tobacco products under their jurisdiction.^17^ Even in Europe, where regulations on ENDs were developed some years ago, regulators have to deal with thousands of notifications each year.^18^ There is a need to set approaches that protect the interests of consumers, public health, particularly with respect to the under-age use of these products, and the environment while allowing innovation to accelerate reductions in cigarette smoking.

There are, of course, also advantages to manufacturers, especially small- to medium-size enterprises, to ensuring that only essential data needs to be collected and submitted to regulators in terms of cost and time, for example, FDA has estimated between $117 000 and $466 000 per pre-market tobacco product application.^19^

In this paper, we look at general regulatory approaches to create these balances and consider how regulations are currently applied in the context of alternative tobacco and nicotine products. The concept of “bridging”—the partial reading across of data sets from an original product to another new but similar product variant—is well established within other industries,^20–22^ and we consider what product development changes in THPs, closed system vaping products (CSVPs) and NPs might be suitable for bridging from existing datasets or trigger the need for new datasets.

## General Approaches to Data Bridging in Consumer Products

There are various reasons why regulators would allow businesses to provide data on modifications of a product without requiring full data packages for each modification. These include improving efficiency in line with regulatory approvals, accepting natural variation in some products, ensuring that innovation that improves products is encouraged and reducing costs to businesses.

The reasons that businesses are driven towards reading across from one dataset to another and using weight-of-evidence approaches to risk assessment are the trends over the past decade of consumers demanding more choice and the responsively increasing speed of innovation.

For sectors that have highly specific sets of regulation (eg, cosmetics), societal factors, such as the need to reduce animal testing, might drive the need for read-across and weight-of-evidence approaches. The EU regulations for cosmetic products state, “The safety of finished cosmetic products can already be ensured based on knowledge of the safety of the ingredients that they contain. Provisions prohibiting animal testing of finished cosmetic products should therefore be laid down. The application, in particular by small- and medium-sized enterprises, of both test methods and assessment procedures for relevant available data, including the use of read-across and weight-of-evidence approaches, which do not involve the use of animals for assessing the safety of finished cosmetic products could be facilitated by Commission guidelines.” ^23^

In other sectors, regulators accept that scientific data pertaining to specific products will have some variability because of natural variation in the products. For example, EU regulations on the tolerances for nutritional labeling of foods note, “The declared values should, according to the individual case, be average values based on the manufacturer’s analysis of the food; a calculation from the known or actual average values of the ingredients used, or a calculation from generally established and accepted data.” ^24^

Depending on the product category, combined approaches are often taken to ensure product safety and quality, consumer protection, and the ability of consumers to make informed and meaningful choices (Panel 1). Regulatory assessment may sit as part of a more holistic regulatory environment in which regulators may perform assessments for a fee and manufacturers are required to apply warnings and restrictions (eg, on age and marketing).

## General Approaches to Data Bridging in Tobacco and Nicotine Consumer Products

For the products that are the focus of this paper—THPs, CSVPs, and NPs—there are evolving regulatory approaches based on their similarities to and differences from traditional tobacco products, their potential impact on population health, the diversity of products available, public health views on tobacco harm reduction, and the potential for these products to reduce projected tobacco-related mortality and morbidity. A report from the WHO Study Group on Tobacco Product Regulation reviews both data and regulatory approaches on THPs and ENDS and makes recommendations for future approaches but does not tackle specifics of data bridging in notification of products.^16^

Currently, the most common of these alternatives to smoking, in terms of geographical and societal uptake, is vaping products. The British Standards Institution (BSI) was the first to publish a guideline on production and testing of e-cigarettes in the vaping product category in 2015.^25^ This guide, which was developed as a consensus standard at around the same time as EU legislation on vaping products was introduced, stated that its purpose was to help businesses, regulators, and consumers. The BSI guideline sets out a requirement on manufacturers to maintain the safety and quality of their products and demonstrate this to regulators, including through ensuring purity of ingredients and measuring potential contaminants from device materials, potential emissions from device operation, and safety of batteries. The guidance also recommends that toxicological assessments should be carried out by a competent person to determine whether additional testing is needed for similar products within a range over that performed on the original product or whether a read-across of results would be appropriate. For example, emissions should be tested proportionately against those from a representative product and a risk-based compliance check used to justify the read-across of the results. A substantial modification to a product requiring a new data set could include, for example, toxicologically significant increases to an ingredient or changes in nicotine concentration greater than 15%.

In 2016, the Medicines and Healthcare products Regulatory Authority (MHRA) became the competent authority on vaping products in the United Kingdom. In its guidance to manufacturers wishing to submit data prior to marketing products, it makes clear that bridging is both possible and appropriate.^14,26^ For example, if a range of nicotine strengths is used, it may be sufficient to test only the highest; if a product is to be sold with a range of options, companies may rely upon data generated from a representative sample and justify choices; or companies may be able to rely on data generated on a subset of flavor or product options on a risk-based basis.

THPs have less of a geographical footprint than CSVPs but have become particularly common in some countries where the latter are greatly restricted, including Japan and South Korea. Under the current EU Tobacco Product Directive, THPs are included in the category Novel Tobacco Products. As with CSVPs and NPs, there is a requirement for notification to the competent authority 6 months prior to marketing, which should provide a range of scientific and consumer behavior information related to the product. Whether and which data sets may be bridged under applicable regulations is less clear for THPs than for CSVPs, although progress has been made on product standards, including scientific tests to determine that the product does not create combustion.^27^

NPs from reputable manufacturers contain food quality flavors and pharmaceutical-grade nicotine in a matrix of microcrystalline cellulose contained in a fleece pouch. These products are relatively new to market. As they are neither tobacco products nor medicinal products, regulatory approaches have varied. NP standards are evolving, and currently include voluntary industry codes and technical product standards to ensure the governance of product safety and quality, including limiting of nicotine content and that nicotine and other ingredients meet pharmaceutical and food quality standards, respectively.^28,29^

## Scientific Principles for Deciding Whether Product Changes are Substantial or May be Bridged

### Closed System Vaping Products

Data for a stable on-market CSVP should be amenable to bridging for a range of nicotine strengths and flavors if a technical dossier has been produced for a product in the range that has the highest nicotine strength and is compliant with applicable emissions and ingredients regulations and standards. However, consideration should be given to whether consumer behavior is different at lower nicotine strengths as compared to higher nicotine strengths. In cigarettes, it was found that smokers switching from higher to lower tar and nicotine products as measured by standardized machine smoking could “compensate” for the lower tar and nicotine by puffing harder or more frequently, with the consequence of a greater toxicant exposure than would have been expected from the testing regime.^30^ Some research suggests that some compensatory behavior may occur with lower nicotine strengths e-liquids and fixed rather than adjustable power devices^31^ and if evidence for such compensation happening then perhaps both highest and lowest strength nicotine liquids should be tested.

Further protection for regulatory interests in some countries (including the EU) is provided in the requirements to report any adverse events experienced by consumers for any product on the market (whether supported by original or bridged data). In Europe, for vaping products, the system is the European Union Rapid Information system (EURapex), which separates vaping products from tobacco and pharmaceuticals^32^ and in the United Kingdom, the MHRA runs a Yellow card scheme for the reporting of any suspected adverse events.^33^

CSVPs come in two parts: first, a device that contains a rechargeable battery and the micro-electronics that control battery usage and supply of power to the heating technology and provide various protections to ensure against overheating; second a consumable cartridge/pod that contains an e-liquid (typically comprising vegetable glycerin and propylene glycol and flavors, with or without nicotine) and the wick and coil, which take up and heat the e-liquid to generate the aerosol. The system parts are designed together to ensure efficient power management and consistent aerosol production throughout the lifetime of the consumable.

All CSVPs should produce substantially lower levels of toxicants than cigarette smoke and incrementally changed products should be amenable to data bridging as long as changes do not: alter the risk of overheating, which could potentially cause thermal degradation of humectants and flavors and generate additional toxicants; use flavors other than those chosen for thermal stability at normal operating temperatures and lack of toxicological impact; or, unfavorably alter the design limits, emissions of metals and interactions between e-liquids and the device. Avoidance of these undesired alterations in performance may be achieved, respectively, by efficient wicking and micro-electronic protections, adhering to regulatory lists of prohibited ingredients,^34^ and requirements to disclose and continue to adhere to metal emissions indicated in the original technical dossier.

For example, in an analysis of one CSVP system conducted by our researchers with different nicotine strengths and flavors,^18,35^ consistent emissions were shown across products for the WHO Study Group on Tobacco Product Regulation toxicants (TobReg 9)^36^ and additional toxicants required to be reported to the UK MHRA (Table 1).^26^ As the WHO Study Group on Tobacco Product Regulation noted^16^ “An ENDS that is truly a ‘closed system’ does not allow the user to alter any of the elements of the device or liquid that influence nicotine yield, e.g. battery voltage, coil resistance, and liquid nicotine concentration; it may also limit user puffing behaviour” though this may not hold true for other types of vaping products such as open systems.

**Table 1.** Emissions for Third- and Fourth-generation CSVPs Versus Combustible Cigarettes

Analyte	Reference cigarette 1R6F^a^	Epen2^b^ 18 mg/mL^d^ BT^e^	Epen3^c^ 18 mg/mL^d^ BT^e^	Epen3^c^ 12 mg/mL^d^ MB^f^, low BA^g^	Epen3^c^ 18 mg/mL^d^ MB^f^, med BA^g^	Epen3^c^ 30 mg/mL^d^ MB^f^, high BA^g^
Carbon monoxide µg/puff	2892	<10.4	<10.4	<10.4	<10.4	<10.4
Formaldehyde µg/puff	4.89	0.27	0.05	0.18	0.19	0.12
Acetaldehyde µg/puff	159	0.23	<0.03	0.10	<0.03	0.03
Acrolein µg/puff	14.5	0.35	<0.01	<0.01	<0.03	0.03
1,3 -butadiene µg/puff	10.0	<0.02	<0.02	<0.02	<0.02	<0.02
Benzene µg/puff	8.65	<0.003	<0.003	<0.003	<0.003	<0.003
Benzo(a)pyrene ng/puff	1.7	<0.00001	<0.00001	<0.00001	<0.00001	<0.00001
NNK ng/puff	21.0	<0.00002	<0.00002	<0.00002	<0.00002	<0.00002
NNN ng/puff	22.7	<0.00001	<0.00001	<0.00001	<0.00001	<0.00001
Aluminum ng/puff	NR^h^	NR	NR	7.7	8.1	3.4
Chromium ng/puff	<0.51	NR	1.8	1.2	1.2	1.5
Iron ng/puff	4.1	NR	2.7	1.3	1.9	4.6
Nickel ng/puff	<1.1	NR	<2.2	<0.25	<2.2	<2.2

^a^University of Kentucky reference cigarette.

^b^Third generation Epen CSVP.

^c^Fourth Generation Epen CSVP.

^d^nicotine concentration in cartridge;

^e^BT = blended tobacco flavor.

^f^MB = Masterblend tobacco flavor.

^g^BA = benzoic acid as an ingredient.

^h^NR = not reported.

Table 1 provides data that show that emissions were lower in the fourth-generation CSVPs than in the third-generation product for analytes sensitive to thermal degradation (formaldehyde, acetaldehyde, and acrolein). Despite the fourth-generation product having higher power, more efficient wicking and micro-electronic controls that reduced the risk of overheating, these analytes were reduced. All other TobReg 9 analytes were either below limits of detection or quantification. Metal emissions for the CSVPs did not differ statistically from air blanks.

Substantial modifications to a CSVP that could trigger the need for additional data would include changes to the way in which the aerosol is created (eg, a variation in wicking system or an increase in power) that might increase toxicant emissions; introduction of new ingredients for which thermal stability or toxicological properties in unheated and heated forms have not been assessed or that might affect degradation of metals in the coil; and increase in the amount of nicotine delivered per puff compared to the nicotine strength of the original product.

### Tobacco Heating Products

Innovation in THPs has been relatively rapid, in terms of both device and consumable format and the way in which the tobacco consumable is heated. The design principles behind THPs are to take specially treated tobacco, typically wrapped in paper to form a rod, and place it in a device that heats it. Humectants in the tobacco rod contribute to an aerosol containing nicotine and flavors.^37^

THPs typically heat tobacco to temperatures less than 350°C, which is sufficient to release the aerosol but avoids combustion. Thus, the production of high levels of multiple toxicants formed by burning cigarettes, which reach temperatures of around 900°C at the peak of the puff, is also avoided. The toxicant profile of THP aerosol is much lower than that of cigarette smoke because many toxicants (eg, carbon monoxide, benzene, and 1,3-butadiene) are either not formed at all or only in trace amounts at the lower temperatures. Those that are formed (eg, tobacco-specific nitrosamines [TSNAs]) are typically emitted at far lower levels than in cigarette smoke.^38^

Although the principles of heating but not burning the tobacco remains similar across different brands of devices, the device formats, methods of heating, and timings of heating differ. All devices contain rechargeable batteries that provide power. Heat sources are either thin-film resistance or induction and micro-electronic controllers create heating profiles that work with the specific tobacco consumable used. The formats and flavors of the consumable tobacco rods also vary, between and within manufacturers. The rods can vary in length and diameter, and ranges of flavors and nicotine strengths are available, but vary to a lesser extent than with CSVPs.

Despite the variability, if the principle of heating and not burning the tobacco is maintained and scientific scrutiny is applied to the flavors used, toxicant profiles of various brands of THPs are all likely to be substantially reduced compared to cigarette smoke. Table 2 presents analytical data on percentage reductions in toxicants from four versions of the same brand of THP compared to cigarette smoke. All have been commercial products in Japan or Korea, and the testing was done using the same tobacco consumable, which was commercially available in Korea. THP1‒3, despite different external appearances (size and weight), are all internally operated in a similar manner using thin-film resistance heating, which heated to around 240°C in 40 s. THP4 used induction heating, allowing a much faster heating time of 20 s, and had a boost function that allowed a higher temperature (maximum 280°C) to be reached in 10 s. The data generally showed similar percentage reductions across THP designs, with slightly lower carbon monoxide reductions but slightly higher TSNA reductions for THP4 in boost mode. This suggests that bridging would be relevant across the four products and three heating conditions.

**Table 2.** Percentage Reductions^a^ in Toxicants for THPs Versus Combustible Cigarettes

Analyte	THP1—originalb	THP2—minic	THP3—nanod	THP4—proe basic function	THP4—pro with boost function^f^
Carbon monoxide	99.5	99.5	99.5	99.4	98.4
Formaldehyde	97.3	97.5	97.5	95.6	95.0
Acetaldehyde	95.8	95.6	95.0	95.0	93.4
Acrolein	99.1	99.1	99.0	98.5	97.9
1,3-butadiene	99.9	99.9	99.9	99.9	99.9
Benzene	99.9	99.9	99.9	99.9	99.9
Benzo(*a*)pyrene	98.4	98.4	98.4	98.4	98.2
NNK^g^	97.2	96.6	96.5	97.3	98.2
NNN^h^	93.9	90.1	89.8	92.4	95.7

^a^Percentage reductions per stick compared with University of Kentucky 1R6F reference cigarette.

^b^Original size, thin-film resistive heating, 40 s to first puff, 3.5 min heating at 240°C.

^c^Smaller size, thin-film resistive heating, 40 s to first puff, 3.5 min heating at 240°C.

^d^Smallest size, thin-film resistive heating, 40 s to first puff, 3.5 min heating at 240°C.

^e^Induction heating, 20 s to first puff, 4.0 min heating at 250°C.

^f^Induction heating, 10 s to first puff, 3 min heating at 280°C (during boost), and then 260°C for a set time.

^g^Nicotine-derived nitrosamine ketone.

^h^ *N*-Nitrosonornicotine. All analytical chemistry was performed at Labstat, Kitchener, ON, Canada using Health Canada Intense smoking conditions with vents unblocked for THPs.

The BSI has published a specification for THPs that should allow some data to be amenable to data bridging.^27^ For example, changes that preserve the operating principle of heating without combustion could be confirmed by the very low levels of combustion markers, such as carbon monoxide and nitrogen oxide. It is suggested that for a product to be accepted as a THP it should have emission levels of carbon monoxide less than 0.3 mg per 100 cm^3^, nitric oxide less than 4 μg per 100 cm^3^, and nitrogen oxides less than 5 μg per 100 cm^3^ under standardized analytical testing conditions.^15,27^ Additional analytes, such as TSNAs, should also be considered for such bridging purposes, as levels should be substantially reduced in the absence of combustion, but standardized analytical methodologies and tolerance ranges remain to be defined. Ingredient selection should maintain the principle of avoiding ingredients of toxicological concern in unheated and heated form.

Substantial modifications that may require additional data collection include changes in heating profile that cause temperatures greater than those set for non-combustion (the limit criteria for carbon monoxide and nitrogen oxide); the use of a novel tobacco substrate with properties that could change the toxicant profile; and the use of technologies that might increase nicotine delivery to above that of a cigarette.

### Nicotine Products

The design principles of NPs are simple. Each NP consists of a fleece pouch that contains a matrix holding pharmaceutical-grade nicotine and flavors, which are emitted steadily over the time of use, and stabilizers. An NP is placed between the upper lip and gum and typically left there for around 30 min and then removed as an intact item.^12^

Box 1 **Summary of regulatory approaches to ensuring product safety and quality**

- Use general products safety approaches in manufacturing and distribution, placing the burden on the manufacturer to apply strong quality control and internal risk assessment approaches, to ensure that anything in the product does not increase its inherent risks, and ensure strong recall procedures.
- Use consensus standards produced under the guidance of national, regional or international standardization organizations and developed with input from manufacturers, regulators and other interested parties, such as public health experts, to ensure a “level playing field” across businesses in a given market, and to ensure any scientific information produced is consistent and meaningful.
- Use product standards that determine certain product features, set limits or prohibit the use of specific ingredients to ensure that all products in a category reach certain standards.
- Set out what tolerances should be in place when products are subject to natural variability.
- Specify what information is required by the regulator as notification prior to placing a product on market or for seeking approval to enter the market.
- Provide advice on what constitutes a substantial modification to an existing product and requires additional data.

NPs are a relatively newly introduced product range that has become popular over the last couple of years in countries typically with a history of oral tobacco use such as Sweden, and science is just beginning to be published on them. Although NPs bear a strong resemblance to Swedish snus in terms of the shape and size of the pouch and packaging and the way that they are used by consumers, they contrast with regard to ingredients. They contain no tobacco and have lower levels of toxicants than snus and a toxicant profile closer to that of nicotine replacement therapy products.^13,39^ Thus, much can be drawn from the extensive chemical, toxicological, clinical, and epidemiological studies of snus. Epidemiological studies on snus show that its exclusive use results in substantially reduced health risks compared to smoking, including decreased incidence of lung cancer, oral cancer, or other respiratory diseases.^4^

A typical range of NPs within a brand and across brands can include various nicotine strengths and flavors.^14^ Unlike CSVPs and THPs, analytical chemical comparisons between NP strengths and flavors are unlikely to differentiate variants and all NPs from reputable manufacturers can be expected to have very low toxicant levels (Table 3). Intake of NP emissions through oral mucosa is qualitatively different than absorption of cigarette smoke through inhalation into the pulmonary system. As such, comparisons are more difficult between cigarette smoke emissions that will impact both the oral cavity and respiratory airways and lung directly, and NPs that are likely to only directly impact the oral mucosa and other parts of the body indirectly through any systemic exposure. However, NPs’ low level of toxicant emissions is comparable and even lower than snus that delivers emissions through the oral mucosa in an identical manner to NPs and has been shown to confer greatly reduced relative risks of disease as compared to conventional cigarettes.^4^

**Table 3.** Percentage Reductions^a^ in Toxicant Emissions for Seven Variants of a Commercial NP Versus Cigarettes

Sample	A	B	C	D	E	F	G
Nicotine level (mg)	4	4	7	10	10	15	20
Weight of pouch (g)	0.8	0.8	0.8	0.8	0.8	1	1.33
Base taste/top flavor complexity	Scan/S^b^	NScan/C^c^	Scan/C^d^	NScan/S^e^	NScan/C^c^	Scan/C^d^	Scan/C^d^
1,3-butadiene	>99.9	>99.9	>99.9	>99.9	>99.9	>99.9	>99.9
Acetaldehyde	>99.9	>99.9	>99.9	>99.9	>99.9	>99.9	>99.9
Acrolein	>99.9	>99.9	>99.9	>99.9	>99.9	>99.9	>99.9
Benzene	>99.9	>99.9	>99.9	>99.9	>99.9	>99.9	>99.9
Benzo(*a*)pyrene	99.1	99.4	99.7	98.9	99.4	99.7	99.0
Formaldehyde	99.5	99.1	99.8	98.0	99.2	99.6	99.6
NNK^f^	99.7	99.7	99.7	99.7	99.7	99.7	99.7
NNN^g^	99.8	99.8	99.8	99.8	99.8	99.8	99.8

^a^Percentage reductions per pouch compared with University of Kentucky 1R6F reference cigarette.

^b^Scandinavian style taste (slightly salty) with a simple flavor mix (eg, mint).

^c^Non-Scandinavian style which is (less salty) with a complex flavor mix (eg, fruit or mint mix).

^d^Scandinavian style taste with a complex flavor mix.

^e^Non-Scandinavian style with a simple flavor mix.

^f^Nicotine-derived nitrosamine ketone.

^g^ *N*-Nitrosonornicotine.

Rapid-throughput toxicological screening, using techniques such as real-time cell analysis, may be helpful in distinguishing between product variants. East et al^22,40^ compared the cytotoxicity of extracts for a range of NP nicotine strengths (4‒10.8 mg per pouch) and flavors, including mint and fruit flavors, from a single manufacturer and compared the data to values for snus. All nicotine strengths and flavors were found to be non-cytotoxic in the acute exposure model, similar to the reference snus product.

All NPs will produce substantially reduced toxicant emissions compared to conventional cigarette smoke, which suggests that data could be amenable to bridging if, for example, the nicotine content and exposure levels do not exceed those of Swedish snus (a limit of 20 mg nicotine per pouch has been suggested), the nicotine and other ingredients meet pharmaceutical and food quality standards, respectively, prohibited substances and ingredients are avoided, and any potential sensitizers or allergens are clearly labeled.

A substantial modification to an NP that might require additional data sets would most likely focus on nicotine delivery. Possible examples are the increased amount or speed of nicotine released through changes in nicotine concentration, formulation, and/or fleece barrier properties.

## Conclusions

A successful tobacco harm reduction approach is likely to accelerate the reduction in cigarette smoking incidence and hence the long-term incidence of smoking-related mortality and morbidity in the population. We believe that the term “successful” combines strong regulatory approaches that protect the interest of consumers and enable innovation by manufacturers to maximize the potential of smokers switching completely to reduced risk tobacco and nicotine alternatives.

In regulatory frameworks that require notification of products, the use of internationally recognized product testing approaches, technical product standards, regulatory limits on key ingredients, and active prohibition of other ingredients could help enable bridging across specific product ranges. This in turn can increase the speed of innovation, foster competition, and limit the burden of assessment for regulators while maintaining product safety (ie, minimizing any inherent risks associated with the products) and quality.

## Supplementary Material

A Contributorship Form detailing each author’s specific involvement with this content, as well as any supplementary data, are available online at https://academic.oup.com/ntr.

ntac041_suppl_Supplementary_Taxonomy-form

## Data Availability

The data underlying this article will be shared on reasonable request to the corresponding author.

## References

[1] Stratton K , Shetty P, Wallace R, Bondurant S, editors. Institute of Medicine Committee to Assess the Science Base for Tobacco Harm Reduction. Clearing the smoke: assessing the science base for tobacco harm reduction. Washington, (DC): National Academy Press (US); 2001.

[2] Eaton DL , Kwan LY, Stratton K, editors. National Academies of Sciences, Engineering, and Medicine; Health and Medicine Division; Board on Population Health and Public Health Practice; Committee on the Review of the Health Effects of Electronic Nicotine Delivery. Public health consequences of e-cigarettes. Washington, DC: National Academies Press (US); 2018. 29894118

[3] McNeill A , Brose LS, Calder R, Bauld L, Robson D. Evidence Review of e-Cigarettes and Heated Tobacco Products 2018: A Report Commissioned by Public Health England. London: Public Health England; 2018.

[4] Royal College of Physicians. Nicotine Without Smoke: Tobacco Harm Reduction. London: Royal College of Physicians; 2016.

[5] McKegany N , Dickson T. Why don’t more smokers switch to using e-cigarettes: the views of confirmed smokers. Int J Environ Res Public Health. 2017;14(6):647. 10.3390/ijerph14060647 PMC5486333 28621763

[6] David. *5 Recent Innovations in Vaping Technology*, www.techgeek365.com; 2020. https://techgeek365.com/5-recent-innovations-in-vaping-technology/. Accessed January 17, 2022.

[7] Trumbo RW , Harper R. Perceived characteristics of e-cigarettes as an innovation by young adults. Health Behav Policy Rev. 2021;2(2):154–162. 10.14485/HBPR.2.2.7 PMC4341835 25729752

[8] Caputi TL . Industry watch: heat-not-burn tobacco products are about to reach their boiling point. Tobacco Control. 2017;26(5):609–610. 10.1136/tobaccocontrol-2016-053264 27558827

[9] Parker-Pope T . *PBS, “Safer” Cigarettes: A History”*. www.PBS.org; 2001, PBS. https://www.pbs.org/wgbh/nova/article/safer-cigarettes-history/. Accessed January 17, 2022.

[10] McGill DC . “‘Smokeless’ Cigarette’s Hapless Start”. The New York Times. ISSN 0362-4331; 1988. https://www.nytimes.com/1988/11/19/business/smokeless-cigarette-s-hapless-start.html. Accessed January 17, 2022.

[11] Mallock N , Pieper E, Hutzler C, Henkler-Stephani F, Luch A. Heated tobacco products, a review of current knowledge and initial assessments. Front. Public Health. 2019;7:1–8. https://doi.org/10.3389/fpubh.2019.00287 10.3389/fpubh.2019.00287 PMC6795920 31649912

[12] Plurphanswat N , Hughes JR, Fagerström K, Rodu B. Initial information on a novel nicotine product. Am J Addict. 2020;29:279–286. 32176374 10.1111/ajad.13020

[13] Patwardhan S , Fagerström K. Nicotine pouches – a research and regulatory policy agenda to maximise public health benefits and minimize harms. Qeios. 2021:1–16. doi:10.32388/L4TIAF.2.

[14] Stanfill S , Tran H, Tyx R, et al. Characterization of total and unprotonated (free) nicotine content of nicotine pouch products. Nic Tob Res. 2021;1590–1596. 10.1093/ntr/ntab030 34233354

[15] Rensch J , Liu J, Wang J, et al Nicotine pharmacokinetics and subjective response among adult smokers using different flavors of on!® nicotine pouches compared to combustible cigarettes. Psychopharmacol. (Berl). 2021;238(11):3325–3334. 10.1007/s00213-021-05948-y 34432106

[16] WHO Study Group on Tobacco Product Regulation. Report on the Scientific Basis of Tobacco Product Regulation: Eighth Report of a WHO Study Group. Geneva: World Health Organization; 2021. (WHO Technical Report Series, No. 1029). 20942227

[17] Zeller M . Perspective: FDA’s Progress on Tobacco Product Application Review and Related Enforcement. 2021. Perspective: FDA’s Progress on Tobacco Product Application Review and Related Enforcement | FDA. www.fda.gov. Accessed January 17, 2022.

[18] UK Medicines and Healthcare Products Regulatory Agency Record of Notifications. *ECIG Search Page* | MHRA. https://cms.mhra.gov.uk/ecig. Accessed January 17, 2022.

[19] Haar MV . An unsettling precedent: what FDA deeming regulations means for retailers and categories beyond vaping. NACS Magazine; 2017. https://www.nacsmagazine.com/issues/january-2017/unsettling-precedent. Accessed January 17, 2022.

[20] European Medicines Agency. *Guideline on Similar Biomedical Medicinal Products*; 2014. https://www.ema.europa.eu/en/documents/scientific-guideline/guideline-similar-biological-medicinal-products-rev1_en.pdf. Accessed January 17, 2022.

[21] ECHA. *Read-across Assessment Framework (RAAF)*. Helsinki: European Chemicals Agency; 2017. https://echa.europa.eu/documents/10162/13628/raaf_en.pdf/614e5d61-891d-4154-8a47-87efebd1851a. Accessed January 17, 2022.

[22] Midha KK , McKay G. Bioequivalence; its history, practice, and future. AAPS J. 2009;11(4):664–70. 19806461 10.1208/s12248-009-9142-z PMC2782076

[23] The European Parliament and the Council and the European Union. Regulation (EC) No 1223/2009 of the European Parliament and of the Council of 30 November 2009 on cosmetic products (Text with EEA relevance). Off J Eur. Union. 2009;L342/59–209. https://ec.europa.eu/health/system/files/2016-11/cosmetic_1223_2009_regulation_en_0.pdf. Accessed January 17, 2022.

[24] European Commission. *Guidance Document for Competent Authorities for the Control of Compliance with EU legislation on: Regulation (EU) No 1169/2011 of the European Parliament and of the Council of 25 October 2011 on the Provision of Food Information to Consumers, Amending Regulations (EC) No 1924/2006 and (EC) No 1925/2006 of the European Parliament and of the Council, and Repealing Commission Directive 87/250/EEC, Council Directive 90/496/EEC, Commission Directive 1999/10/EC, Directive 2000/13/EC of the European Parliament and of the Council, Commission Directives 2002/67/EC and 2008/5/EC and Commission Regulation (EC) No 608/2004 and Council Directive 90/496/EEC of 24 September 1990 on Nutrition Labelling of Foodstuffs and Directive 2002/46/EC of the European Parliament and of the Council of 10 June 2002 on the Approximation of the Laws of the Member States Relating to Food Supplements with Regard to the Setting of Tolerances for Nutrient Values Declared on a Label*; 2012. labelling_nutrition-vitamins_minerals-guidance_tolerances_1212_en.pdf. Accessed January 17, 2022.

[25] BSI. *PAS 54115:2015. Vaping Products, Including Electronic Cigarettes, e-Liquids, e-Shisha and Directly-related Products – Manufacture, Importation, Testing and Labelling. Guide*. London: British Standards Institution; 2015.

[26] Medicines and Healthcare Products Regulatory Agency. *E-cigarettes: Regulations for Consumer Products. Guidance on How to Get an e-Cigarette on the Market in Great Britain and Northern Ireland, Including the Notification Scheme and Reporting Problems with e-Cigarettes*; 2016. https://www.gov.uk/guidance/e-cigarettes-regulations-for-consumer-products. Accessed January 17, 2022.

[27] BSI. PAS 8850:2020 Non-combusted tobacco products. Heated tobacco products and electrical tobacco heating devices. Specification. London: British Standards Institution; 2020.

[28] Svenska Institutet för Standarer. *Nicotine-containing, Tobacco-Free Oral Products - Safety and Quality-related Requirements*; 2020. https://www.sis.se/produkter/jordbruk-0faca7db/tobak-tobaksprodukter-och-tillhorande-utrustning/sists-722020/. Accessed January 17, 2022.

[29] Pakistan Standards. *PS/ 5468-2020: Nicotine Containing Tobacco Free Oral Product*. http://updated.psqca.com.pk/oral/. Accessed January 17, 2022.

[30] US National Cancer Institute. *Risks Associated with Smoking Cigarettes with Low Machine-Measured Yields of Tar and Nicotine. Tobacco Control Monograph No. 13*. Bethesda, MD: U.S. Department of Health and Human Services, National Institutes of Health, National Cancer Institute; 2001.

[31] Cox S , Goniewicz ML, Kosmider L, McRobbie H, Kimber C, Dawkins L. The time course of compensatory puffing with an electronic cigarette: secondary analysis of real-world puffing data with high and low nicotine concentration under fixed and adjustable power settings. Nicotine Tob Res. 2021;23(7):1153–1159. 33483754 10.1093/ntr/ntab013 PMC8186419

[32] European Rapid Alert System for Dangerous Products (RAPEX). https://ec.europa.eu/safety-gate-alerts/. Accessed January 17, 2022.

[33] Drug Safety Update. E-cigarette Use or Vaping: Reporting Suspected Adverse Events, Including Lung Injury, 27 January 2020. E-cigarette use or vaping: reporting suspected adverse reactions, including lung injury - GOV.UK. www.gov.uk. Accessed January 17, 2022.

[34] *EU Directive 2014/40 of the European Parliament and of the Council of 3 April 2014 on the Approximation of the Laws, Regulations and Administrative Provisions of the Member States Concerning the Manufacture, Presentation and Sale of Tobacco and Related Products* . Luxembourg: Office for Official Publications of the European Communities; 2014. L 127/1-38. https://www.legislation.gov.uk/eudr/2014/40/pdfs/eudr_20140040_adopted_en.pdf. Accessed January 17, 2022.

[35] Cunningham A , McAdam K, Thissen J, Digard H. The evolving e-cigarette: comparative chemical analyses of e-cigarette vapor and cigarette smoke. Front Toxicol. 2020;2:1–24. doi:10.3389/ftox.2020.586674. 10.3389/ftox.2020.586674 PMC8915913 35296117

[36] Burns D , Dybing E, Gray N, et al Mandated lowering of toxicants in cigarette smoke: a description of the World Health Organization TobReg proposal. Tob Control. 2008;17(2):132–141. 18375736 10.1136/tc.2007.024158 PMC2569138

[37] Eaton D , Jakaj B, Forster M, et al Assessment of tobacco heating product THP1.0. part 2: product design, operation and thermophysical characterisation. Regul Toxicol Pharmacol. 2018;93:4–13. 29080851 10.1016/j.yrtph.2017.09.009

[38] Forster M , Fiebelkorn S, Yurteri C, et al Assessment of novel tobacco heating product THP 1.0. Part 3: comprehensive chemical characterisation of harmful and potentially harmful aerosol emissions. Regul Toxicol Pharmacol. 2018;93:14–33. 29080848 10.1016/j.yrtph.2017.10.006

[39] Azzopardi D , Liu C, Murphy J, Chemical characterisation of tobacco-free “modern” oral nicotine pouches and their position on the toxicant and risk continuums. Drug Chem Toxicol. 2021. doi: 10.1080/01480545.2021.1925691. 10.1080/01480545.2021.1925691 34034614

[40] East N , Bishop E, Breheny D, Gaca M, Thorne DA. screening approach for the evaluation of tobacco-free “modern oral” nicotine products using real time cell analysis. Toxicol Rep. 2021;8:481–488. 33718000 10.1016/j.toxrep.2021.02.014 PMC7933807

